# Security Analysis and Improvement of ‘a More Secure Anonymous User Authentication Scheme for the Integrated EPR Information System’

**DOI:** 10.1371/journal.pone.0131368

**Published:** 2015-08-11

**Authors:** SK Hafizul Islam, Muhammad Khurram Khan, Xiong Li

**Affiliations:** 1 Department of Computer Science and Information Systems, Birla Institute of Technology and Science, Pilani, Rajasthan 333031, India; 2 Center of Excellence in Information Assurance, King Saud University, Riyadh, Saudi Arabia; 3 School of Computer Science and Engineering, Hunan University of Science and Technology, Hunan 411201, Xiangtan, China; Nankai University, CHINA

## Abstract

Over the past few years, secure and privacy-preserving user authentication scheme has become an integral part of the applications of the healthcare systems. Recently, Wen has designed an improved user authentication system over the Lee et al.’s scheme for integrated electronic patient record (EPR) information system, which has been analyzed in this study. We have found that Wen’s scheme still has the following inefficiencies: (1) the correctness of identity and password are not verified during the login and password change phases; (2) it is vulnerable to impersonation attack and privileged-insider attack; (3) it is designed without the revocation of lost/stolen smart card; (4) the explicit key confirmation and the no key control properties are absent, and (5) user cannot update his/her password without the help of server and secure channel. Then we aimed to propose an enhanced two-factor user authentication system based on the intractable assumption of the quadratic residue problem (QRP) in the multiplicative group. Our scheme bears more securities and functionalities than other schemes found in the literature.

## 1 Introduction

Due to the rapid progress of the communication technologies and information security, anonymous and secure remote user mutual authentication schemes are widely employed in the integrated electronic patient record (EPR) information system [1–8]. The online services provided by the EPR information system not only save patient’s valuable time, but also helps doctor to take correct and quick clinical decision based on the digital information available on the remote location server of EPR information system [9–11]. In addition, with this online facility, the patient residing at home can access his/her confidential health report [12–15] stored on the EPR server through the internet. On the other hand, the doctor can access and analyze patient’s data and can also inform the patients in a timely manner. Accordingly, to provide such type of facilities to the patients and the doctors, many healthcare systems are now being replaced the traditional paper-based system with digital service over wireless networks [16–21]. However, the privacy and confidentiality of patient health data must be maintained when these are accessed from the server over the internet [22–24]. Since the internet is ubiquitous in nature and thus the malicious adversaries may try to collect the confidential data of the patients. Thus, a robust and flexible authentication scheme usable in integrated EPR information system is required to access patient’s data over any insecure channel [25–29].

### 1.1 Related Works

In order to maintain the security and privacy of the integrated EPR information system, many smart card based (two-factor) user authentication systems have been presented recently [2, 3, 30–34]. However, it has been analyzed from the security community that the previous schemes are no longer provide required security and functional requirements of a robust user authentication system [12, 13, 17, 25, 28, 29]. In 2012, Wu et al. [32] devised a new user authentication scheme for the integrated EPR information system with password and smart card. Then they argued that their scheme [32] resists all the vulnerabilities and includes all the functionalities. However, Lee et al. [2] demonstrated that Wu et al.’s scheme [32] is incapable to resist the stolen verifier attack and the lost smart card attack. Then an improved scheme was proposed by them and claimed that the scheme [2] is strong enough to remove the known vulnerabilities. Recently, Wen [3] has adopted the intractability assumption of the quadratic residue problem (QRP) [35, 36] and designed an enhanced scheme over the scheme proposed by Lee et al. [2].

### 1.2 Motivation and Contribution

The Wen’s scheme [3] is analyzed to be secure and efficient than other schemes [2, 35], however, this paper identifies many inefficiencies on Wen’s scheme [3]. This paper carefully analyzed that Wen’s scheme [3] is suffering from the following problems: (1) it can not check user identity and password in the login and password change phases; (2) it is weak against the impersonation attack and privileged-insider attacks; (3) it has no facility to revoke the lost/stolen smart card; (4) the *explicit key confirmation* and the *no key control* properties of the session key are absent, and (5) it does not update user password without any secure channel and the remote server’s assistance. In this study, the authors have considered all the security and functionality features, and consequently presented a new user authentication scheme, which is suitable for the application of integrated EPR information system. The performance studies have proved that the proposed design has eliminated the pitfalls of Wen’s scheme [3]. Our user authentication would be more applicable for healthcare applications, such as the integrated EPR information system.

### 1.3 Organization of the Paper

We organized the rest parts of this paper as follows. The quadratic residue problem required to understand the rest of this paper is explained in Section 2. We explained the Wen’s authentication scheme in Section 3. The weaknesses of Wen’s scheme are presented in Section 4. The improved scheme is proposed in Section 5, and its security and functionality discussions are proposed in Section 6. The Section 7 provides a comparative analysis and the Section 8 concludes the paper.

## 2 Quadratic Residue Problem

In this section, we briefly introduce the quadratic residue problem (QRP) [36]. Suppose that the composite number *n* is the product of two large prime numbers *p* and *q*. We can say that *b* is a quadratic residue modulus *n*, if the equation *b* ≡ *a* ^2^ mod *n* is solvable in the multiplicative group $Z_{n}^{*}$. The set of quadratic residue modulo *n* is defined as

$$\begin{array}{ccc} QR_{n} & = & \left\{b:\exists \;a\in Z_{n}^{*}\;\text{such}\;\text{that}\;b\equiv a^{2}\;\text{mod}\;n\right\} \end{array}$$ (1)

In cryptography, many schemes/protocols [3, 35] are designed under the intractability assumption of the QRP. The hardness assumption of QRP is equivalent to the factoring the large modulus *n*. That is for given *b* ∈ *QR*_*n*_, it is infeasible for a polynomial time bounded algorithm to find *a* without factoring the public modulus *n*.

## 3 Description of the Wen’s User Authentication Scheme

Here, we present Wen’s two-factor user authentication scheme [3]. Initially, the remote medical server *S* selects two large prime numbers *p*, *q* and calculates the modulus as *n* = *pq*. Now, *S* made public *n*, whereas *p* and *q* are kept secret. The list of notations needed to understand the later part of the paper are described in Table 1.

**Table 1:** Descriptions of various notations.

Notations	Description
*U*_*i*_	The patient (User)
*ID*_*i*_	The identity of *U*_*i*_
*PW*_*i*_	The password of *U*_*i*_
*S*	The medical server of integrated EPR information system
*K*	The secret key of *S*
*c*_*i*_	The counter maintained in *S*’s database against the user *U*_*i*_
*H*(⋅)	The secure and collision-resistance one-way hash function
*p*, *q*	Two large prime numbers
*n*	The publicly known modulus such that *n* = *pq*
$b\;\in_{R}\;Z_{n}^{*}$	The number *b* randomly chosen from $Z_{n}^{*}$
⊕	The bitwise XOR operator
∥	The concatenation operator
*SK*	The session key agreed between *U*_*i*_ and *S*
𝓐	The active/passive adversary

The following phases are used to described Wen’s authentication scheme.

### 3.1 Registration Phase

In this phase, the patient *U*_*i*_ performs the registration through secure channel to the integrated EPR information server *S* in order to obtain a valid smart card. We explained this phase by the following steps:

**Step 1**. *U*_*i*_ sends a registration request with his/her 〈*ID*_*i*_, *PW*_*i*_〉 to *S* over a secure channel.
**Step 2**. *S* verifies the correctness of *ID*_*i*_ and computes *v* = *H*(*K*⊕*ID*_*i*_).
**Step 3**. *S* calculates *s*_1_ = *H*(*PW*_*i*_∣∣*K*), *s*_2_ = *H*(*H*(*PW*_*i*_∣∣*s*_1_)) and *N* = *v*⊕*s*_2_.
**Step 4**. *S* initiates a counter *c*_*i*_ = 0 against *U*_*i*_ and insert a tuple 〈*ID*_*i*_, *c*_*i*_〉 in the database. Then *S* issues a new smart card against *U*_*i*_ that includes the information 〈*H*(⋅), *N*, *s*_1_, *c*_*i*_〉.
**Step 5**. *S* sends the smart card to *U*_*i*_ over a secure channel.

### 3.2 Login Phase

For the login purpose, *U*_*i*_ performs the following steps:

**Step 1**. *U*_*i*_ inserts the smart card into the specific card reader and keys his/her 〈*ID*_*i*_, *PW*_*i*_〉. Smart card then selects a number *r* and computes *s*_2_ = *H*(*H*(*PW*_*i*_∣∣*s*_1_)).
**Step 2**. Smart card computes *c*_*i*_ = *c*_*i*_ + 1 and *M*_1_ = (*ID*_*i*_∣∣*N*∣∣*s*_2_∣∣*r*∣∣*c*_*i*_)^2^ mod *n*. Now, *U*_*i*_ sends *M*_1_ as a login message to *S* over a public network.

### 3.3 Authentication Phase

In this phase, the user (patient) *U*_*i*_ and the server *S* perform mutual authentication and then agreed on common secret session key. The description of this phase is given with the following steps:

**Step 1**. When *S* received *M*_1_, then extracts (*ID*_*i*_∣∣*N*∣∣*s*_2_∣∣*r*∣∣*c*_*i*_) from *M*_1_ based on Chinese Remainder Theorem (CRT) using the secret primes *p* and *q*. Now, *S* takes the tuple 〈*ID*_*i*_, $c_{i}^{\prime }\rangle$ from his/her own database and verifies whether $c_{i}^{\prime }>c_{i}$ holds. If $c_{i}^{\prime }>c_{i}$ is incorrect, then *S* aborts the session. Otherwise, *S* updates the tuple 〈*ID*_*i*_, $c_{i}^{\prime }\rangle$ to 〈*ID*_*i*_, *c*_*i*_〉 and continues to the next steps.
**Step 2**. *S* computes *v* = *H*(*K*⊕*ID*_*i*_), $s_{2}^{\prime }=N⊕v$ and verifies it with *s*_2_. If $s_{2}^{\prime }=s_{2}$, *S* then accepts *U*_*i*_ as an legitimate user. *S* computes the session key *SK* = *H*(*s*_2_∣∣*r*∣∣1).
**Step 3**. *S* also performs the calculation of the response message as *M*_2_ = *H*(*s*_2_∣∣*r*∣∣0) and then sends it to *U*_*i*_ over a public network.
**Step 4**. When *U*_*i*_ received *M*_2_, then computes $M_{2}^{\prime }$ = *H*(*s*_2_∣∣*r*∣∣0) and checks whether $M_{2}^{\prime }$ = *M*_2_ is correct or not. If $M_{2}^{\prime }\neq M_{2}$, *U*_*i*_ aborts the session. Otherwise, *U*_*i*_ authenticates *S* and computes the session key *SK* = *H*(*s*_2_∣∣*r*∣∣1).

The complete description of login phase and authentication phase of Wen’s scheme [3] is further presented in Table 2.

**Table 2:** Login and Authentication phases of Wen’s scheme [3].

**User *U*_*i*_/Smartcard**		**Server *S***
**User *U*_*i*_**:		
Insert ⟨*ID*_*i*_, *PW*_*i*_⟩		
**Smartcard**:		
Select a random number *r*		
Compute *s*_2_ = *H*(*H*(*PW*_*i*_∣∣*s*_1_), *c*_*i*_ = *c*_*i*_ + 1		
Compute *M*_1_ = (*ID*_*i*_∣∣*N*∣∣*s*_2_∣∣*r*∣∣*c*_*i*_)^2^ mod *n*		
	$\overset{M_{1}}{\underset{\left(\text{via a public channel}\right)}{\to }}$	
		Extract ⟨*ID*_*i*_, *N*, *s*_2_, *r*, *c*_*i*_⟩ from *M*_1_
		Obtain ⟨*ID*_*i*_, $c_{i}^{\prime }\rangle$ from database
		If ($c_{i}^{\prime }\leq c_{i}$)
		abort the session
		Else
		update ⟨*ID*_*i*_, $c_{i}^{\prime }\rangle$ to ⟨*ID*_*i*_, *c*_*i*_⟩
		Compute *v* = *H*(*K*⊕*ID*_*i*_), $s_{2}^{\prime }=N⊕v$
		If ($s_{2}^{\prime }\neq s_{2}$)
		abort the session
		Else
		authenticate *U*_*i*_
		Compute the session key *SK* = *H*(*s*_2_∣∣*r*∣∣1)
		Compute *M*_2_ = *H*(*s*_2_∣∣*r*∣∣0)
	$\overset{M_{2}}{\underset{\left(\text{via a public channel}\right)}{\leftarrow }}$	
**Smartcard**:		
Compute $M_{2}^{\prime }$ = *H*(*s*_2_∣∣*r*∣∣0)		
If ($M_{2}^{\prime }$ *M*_2_)		
abort the session		
Else		
authenticate *S*		
Compute the session key *SK* = *H*(*s*_2_∣∣*r*∣∣1)		

### 3.4 Password Change Phase

This phase is executed by *U*_*i*_ and in the cooperation of *S*. By this phase, *U*_*i*_ is allowed to update his/her old password to a new password with the following operations:

**Step 1**. *U*_*i*_ delivers his/her 〈*ID*_*i*_, *PW*_*i*_, *PW*_*new*_〉 to *S* using a secure channel.
**Step 2**. *S* computes *v* = *H*(*K*∣∣*ID*_*i*_), $s_{1}^{*}$ = *H*(*PW*_*new*_∣∣*K*), $s_{2}^{*}=H(H(PW_{new}||s_{1}^{*}))$ and $N^{*}=v⊕s_{2}^{*}$. *S* then securely sends $\langle s_{1}^{*}$, N*〉 to *U*_*i*_. On receiving $\langle s_{1}^{*}$, N*〉 from *S*, *U*_*i*_ updates the old smart card’s memory as 〈*ID*_*i*_, *H*(⋅), N*, $s_{1}^{*}\rangle$.

**Note**: In the password change phase of Wen’s scheme [3], the counter *c*_*i*_ is not incorporated in the smart card and we consider it as typo. Here we assumed that *S* sends the tuple $\langle s_{1}^{*}$, N*〉 to *U*_*i*_ and then *U*_*i*_ updates the old smart card’s memory to 〈*H*(⋅), N*, $s_{1}^{*}$, *c*_*i*_〉.

## 4 Security Pitfalls of Wen’s Authentication Scheme

This section is presented to identify and analyze the security and design issues of Wen’s authentication scheme [3]. The following problems have been observed and their detailed descriptions are given below:

### 4.1 Login Phase is Inefficient and Unfriendly

We claimed that the design of login phase of Wen’s authentication scheme is inefficient and unfriendly. In this phase, *U*_*i*_ keys his/her 〈*ID*_*i*_, *PW*_*i*_〉 into the smart card and then the smart card computes the login message *M*_1_ without verifying the correctness of the entered login identity *ID*_*i*_ and the password *PW*_*i*_. If *U*_*i*_ incorrectly enters the login identity and the password by mistake, then the smart card computes the incorrect login message *M*_1_ and then transfer it to *S*. On receiving *M*_1_, *S* checks it and accordingly informs *U*_*i*_. Therefore, the correctness of 〈*ID*_*i*_, *PW*_*i*_〉 will be checked by *S* not by the smart card. However, this kind of design puts unnecessary burden on *S*. In the literature, efficient smart card based authentication schemes are proposed [28, 29, 37] where instead of *S*, the smart card is responsible for checking the correctness of 〈*ID*_*i*_, *PW*_*i*_〉 before computing the login message *M*_1_. The complete description of the problem we pointed out in Wen’s scheme [3] is explained as follows:

**Case 1**: Here we will show how the login and authentication phases will faced problem if *U*_*i*_ mistakenly insert the incorrect login identity $ID_{i}^{*}$ instead of the correct identity *ID*_*i*_.
**Step 1**. *U*_*i*_ enters $\langle ID_{i}^{*}$, *PW*_*i*_〉 into the smart card and then the smart card selects a random number *r* and calculates *s*_2_ = *H*(*H*(*PW*_*i*_∣∣*s*_1_), *c*_*i*_ = *c*_*i*_ + 1 and *M*_1_ = ${\left(ID_{i}^{*}||N||s_{2}||r||c_{i}\right)}^{2}$ mod *n*. The smart card then sends *M*_1_ to *S* over a public channel.
**Step 2**. In the authentication phase, *S* extracts $\left(ID_{i}^{*}||N||s_{2}||r||c_{i}\right)$ from *M*_1_ based on the Chinese Remainder Theorem (CRT) using the secret primes *p* and *q*. Now, *S* observed that $ID_{i}^{*}$ is incorrect by comparing it with the tuples 〈*ID*_*i*_, $c_{i}^{\prime }\rangle$ stored in the database and accordingly he/she aborts the session.
**Case 2**: Now we show that the login and authentication phases of Wen’s scheme [3] will suffer from the problem as described below if *U*_*i*_ mistakenly insert the incorrect password $PW_{i}^{*}$ instead of the correct password *PW*_*i*_.
**Step 1**. *U*_*i*_ enters 〈*ID*_*i*_, $PW_{i}^{*}\rangle$ into the smart card and then the smart card selects a random number *r* and calculates $s_{2}^{*}=H(H(PW_{i}^{*}||s_{1})$, *c*_*i*_ = *c*_*i*_ + 1 and *M*_1_ = ${\left(ID_{i}||N||s_{2}^{*}||r||c_{i}\right)}^{2}$ mod *n*. The smart card then sends *M*_1_ to *S* over a public network.
**Step 2**. In the authentication phase, *S* extracts $\left(ID_{i}||N||s_{2}^{*}||r||c_{i}\right)$ from *M*_1_ based on the Chinese Remainder Theorem (CRT) using the secret primes *p* and *q*. In this case, *S* found that *ID*_*i*_ and the condition $c_{i}^{\prime }>c_{i}$ are correct and thus performs additional verifications. Then *S* computes *v* = *H*(*K*⊕*ID*_*i*_) and
    $$\begin{array}{ccc} s_{2}^{\prime } & = & N⊕v\\ & = & v⊕s_{2}⊕v\\ & = & s_{2}\\ & = & H(H(PW_{i}\left|\right|s_{1}))\\ & \neq & s_{2}^{*}[\text{Since}\;H(H(PW_{i}^{*}\left|\right|s_{1})\neq H(H(PW_{i}\left|\right|s_{1})] \end{array}$$
    Although, *U*_*i*_ is a legal user, however, *S* rejects him/her since the verification equation $s_{2}^{\prime }$ = $s_{2}^{*}$ is not satisfied.

From the above discussions, we can assured that an efficient and robust authentication scheme must verifies the login identity and password before proceed to the authentication phase.

### 4.2 Password Change Phase is Inefficient and Unfriendly

The password change phase of Wen’s scheme [3] is also inefficient and unfriendly [28, 29, 37]. In this phase, *U*_*i*_ sends his/her 〈*ID*_*i*_, *PW*_*i*_, *PW*_*new*_〉 to *S* through a secure channel. *S* computes *v* = *H*(*K*∣∣*ID*_*i*_), $s_{1}^{*}$ = *H*(*PW*_*new*_∣∣*K*), $s_{2}^{*}=H(H(PW_{new}||s_{1}^{*}))$ and $N^{*}=v⊕s_{2}^{*}$. *S* then securely sends $\langle s_{1}^{*}$, N*〉 to *U*_*i*_. On receiving $\langle s_{1}^{*}$, N*〉 from *S*, *U*_*i*_ updates the smart card’s memory as 〈*ID*_*i*_, *H*(⋅), N*, $s_{1}^{*}$, *c*_*i*_〉. However, the following inefficiencies have been observed in Wen’s scheme:

**Case 1**: The user *U*_*i*_ must used a secure channel to deliver 〈*ID*_*i*_, *PW*_*i*_, *PW*_*new*_〉 to *S* and *S* also used the secure channel to send $\langle s_{1}^{*}$, N*〉 to *U*_*i*_. However, each and every password change, Wen’s scheme [3] needs two secure communication channels and it is costly and difficult to achieve in real environments. As a result, *U*_*i*_ will not be interested to change his/her password periodically. However, due to the security reasons, it is recommended to change the password periodically.
**Case 2**: For the password change operation of smart card based authentication scheme, it is recommended that the smart card itself change the password without any connection with the remote server *S* [37].
**Case 3**: During the password change, the correctness of the entered old identity and password 〈*ID*_*i*_, *PW*_*i*_〉 must be verified before changing the old password *PW*_*i*_ to a new password *PW*_*new*_.

However, all of the aforesaid conditions are not incorporated in the password change phase of the Wen’s authentication scheme [3].

### 4.3 Impersonation Attack

In Wen’s scheme, the old password *PW*_*i*_ has no role in the password change operation. Therefore, if the adversary chooses two random passwords $PW_{i}^{\prime }$ and $PW_{i}^{\prime \prime }$, and issues a password change request on behalf of *U*_*i*_ to *S* by sending 〈*ID*_*i*_, $PW_{i}^{\prime }$, $PW_{i}^{\prime \prime }\rangle$. Upon receiving the password change request, *S* computes *v* = *H*(*K*∣∣*ID*_*i*_), $s_{1}^{\prime \prime }$ = $H(PW_{i}^{\prime \prime }||K)$, $s_{2}^{\prime \prime }=H(H(PW_{i}^{\prime \prime }||s_{1}^{\prime \prime }))$ and $N^{\prime \prime }=v⊕s_{2}^{\prime \prime }$, and sends $\langle s_{1}^{\prime \prime }$, *N*′′〉 to the adversary. Upon receiving $\langle s_{1}^{\prime \prime }$, *N*′′〉, the adversary chooses a sufficiently large value $c_{i}^{\prime \prime }$ as the counter and then stores the tuple 〈*ID*_*i*_, *H*(⋅), *N*′′, $s_{1}^{\prime \prime }$, $c_{i}^{\prime \prime }\rangle$ into a smart card. It can be noted that, the adversary can successfully impersonate *U*_*i*_ by using this smart card and 〈*ID*_*i*_, $PW_{i}^{\prime \prime }\rangle$.

### 4.4 Privileged-Insider Attack

It is difficult for a user to remember a number of passwords, if he/she registers himself/herself to different applications or servers with different passwords [37, 38]. Therefore, it is common in real-life environments that a user accesses a number of servers with the common password and identity. However, if the password of the user is known by some means to the privileged-insider of a server, then of course he may try to impersonate the user to access other application servers. We can define the insider attacker is any manager of the authentication server, whose intention is to leak the secret information leading to compromise the system. In the registration phase of Wen’s authentication scheme [3], *U*_*i*_ transmitted 〈*ID*_*i*_, *PW*_*i*_〉 in plaintext form to *S*, then the malicious privileged-insider of *S* may impersonate *U*_*i*_ by login to other application servers using the known 〈*ID*_*i*_, *PW*_*i*_〉. Therefore, Wen’s authentication scheme is not secure against privileged-insider attack.

### 4.5 Absence of Lost/Stolen Smart Card Revocation Phase

In a smart card based authentication scheme, the revocation of lost/stolen smart card plays a vital role in order to provide the adequate security to the end user [39]. However, Wen’s scheme [3] has not offered such an important security features. In the design of two-factor user authentication system, most of the researchers offers a realistic assumption that the smart card is non-temper resistance. It includes that if an adversary obtains a smart card, then he/she can perform some off-line analysis by monitoring the timing information, power consumption and reverse engineering techniques as presented in [40–42] and can obtain the information from the lost smart card. Now the adversary may apply some off-line procedure on the extracted information and may get success to find the correct password of the user. If the adversary found the correct password, then he/she can masquerade the corresponding legal user by using the guessed password and the lost smart card [37–39]. Thus, the cryptographic research community suggested that the lost/stolen smart card revocation phase must be incorporated in two-factor user authentication scheme so that the remote server can distinguish the lost smart card and the new smart card.

### 4.6 Absence of Explicit Session Key Confirmation Property

According to the analysis provided in [43], an authenticated key agreement (AKA) scheme must have the explicit session key confirmation (implicit key authentication and key confirmation) property. The implicit key conformation property includes that the user *X* is assured that *Y* can compute the session key. However, the explicit key confirmation property states that the user *X* is assured that the user *Y* has actually computed the session key. Therefore, only the explicit key confirmation property provides the stronger assurances that *X* and *Y* hold the same session key. A key agreement scheme that includes explicit key authentication is termed as authenticated key agreement with key confirmation (AKC) scheme. In the authentication phase of Wen’s scheme [3], *S* computes *M*_2_ = *H*(*s*_2_∣∣*r*∣∣0) and sends it to *U*_*i*_. On receiving *M*_2_, *U*_*i*_ computes $M_{2}^{\prime }$ = *H*(*s*_2_∣∣*r*∣∣0) and authenticates *S* if the verification $M_{2}^{\prime }$ = *M*_2_ is correct. After this verification, *U*_*i*_ computes the session key as *SK* = *H*(*s*_2_∣∣*r*∣∣1). As the authentication message *M*_2_ does not include the session key information, therefore, the explicit session key confirmation property is not achieved in Wen’s authentication scheme.

### 4.7 Absence of No Key Control Property

The no key control property of an AKA scheme means that none of the users have control over others [38, 43, 44]. That is none of the users or even an adversary can force other so that the session key to be a pre-selected value or it may lie within a set consisting of small number of elements. Thus, we can say that an AKA scheme has the no key control property if the session key is computed with the contributions of all the participants. In Wen’s scheme [3], we observed that the final session key agreed between *U*_*i*_ and *S* is *SK* = *H*(*s*_2_∣∣*r*∣∣1), where *U*_*i*_ chooses the random number *r*. Now it is clear that *S* has no contribution on the session key. Therefore, the *no key control* property is absent in Wen’s authentication scheme.

## 5 The Proposed User Authentication Scheme

In the following, an improved user authentication scheme is presented that not only eliminates the inefficiencies of Wen’s authentication scheme [3], but also includes additional security and functional properties of a two-factor authentication scheme. Similar to the Wen’s scheme, the security of our user authentication scheme is based on the intractable assumption of the quadratic residue problem (QRP) in the multiplicative group $Z_{n}^{*}$ [3, 35, 36]. Initially, the remote server *S* of the integrated EPR information system selects two large prime numbers *p*, *q*. *S* discloses the public modulus *n*, whereas *p* and *q* are kept secret from the outsiders. *S* also selects $K\;\in_{R}\;Z_{n}^{*}$ as his/her secret (private) key. Our enhanced scheme includes the following phases, called *registration phase*, *login phase*, *authentication phase*, *password change phase* and *lost/stolen smart card revocation phase*. The complete explanation of these phases are given below.

### 5.1 Registration Phase

In this phase, a legal user *U*_*i*_ registerers himself/herself to the remote server *S* and obtains a valid medical smart card from *S*. The following steps are executed by *U*_*i*_ and *S*:

**Step 1**. *U*_*i*_ issues a registration request with his/her identity *ID*_*i*_ to *S* over a secure channel.
**Step 2**. On receiving *ID*_*i*_, *S* checks whether *ID*_*i*_ is fresh or not. If it is found in the *S*’s database, then *S* informs *U*_*i*_ to supply a fresh login identity. Otherwise, *S* selects a number $b_{i}\;\in_{R}\;Z_{n}^{*}$ and then computes *A*_*i*_ = *H*(*ID*_*i*_∣∣*K*∣∣*b*_*i*_). After that, *S* initiates a counter *c*_*i*_ = 0 [3] and selects a new smart card that includes the information 〈*H*(⋅), *n*, *A*_*i*_, *c*_*i*_〉. Then *S* securely delivers the smart card to the user *U*_*i*_. *S* includes the tuple 〈*ID*_*i*_, *c*_*i*_, *b*_*i*_〉 into the database [45].
**Step 3**. On receiving the smart card, *U*_*i*_ inserts the smart card into the card reader and keys his/her login identity *ID*_*i*_ and the password *PW*_*i*_ into the smart card. Then the smart card computes *B*_*i*_ = *H*(*ID*_*i*_∣∣*PW*_*i*_), *C*_*i*_ = *A*_*i*_⊕*B*_*i*_ and *D*_*i*_ = *H*(*A*_*i*_∣∣*B*_*i*_). Now the smart card deletes the information 〈*A*_*i*_, *B*_*i*_〉 from the memory and then updates it by the tuple 〈*H*(⋅), *n*, *C*_*i*_, *D*_*i*_, *c*_*i*_〉.

### 5.2 Login Phase

In this phase, *U*_*i*_ computes a login message and sends it to *S* for verification. The login phase includes the following steps:

**Step 1**. *U*_*i*_ inserts the smart card into the specific card reader and keys his/her 〈*ID*_*i*_, *PW*_*i*_〉 into the smart card. The smart card computes *B*_*i*_ = *H*(*ID*_*i*_∣∣*PW*_*i*_), *A*_*i*_ = *C*_*i*_⊕*B*_*i*_ and $D_{i}^{\prime }$ = *H*(*A*_*i*_∣∣*B*_*i*_). The smart card aborts the login process if $D_{i}^{\prime }\neq D_{i}$ holds. Otherwise, the smart card executes the following steps.
**Step 2**. The smart card computes *c*_*i*_ = *c*_*i*_ + 1 and the login message *M*_1_ = (*ID*_*i*_∣∣*A*_*i*_∣∣*a*∣∣*c*_*i*_)^2^ mod *n*, where the number $a\;\in_{R}\;Z_{n}^{*}$ is chosen by the smart card. Then the smart card sends *M*_1_ to *S* over a public network.

### 5.3 Authentication Phase

**Step 1**. Upon receiving *M*_1_, *S* then obtains (*ID*_*i*_∣∣*A*_*i*_∣∣*a*∣∣*c*_*i*_) from *M*_1_ using the Chinese Remainder Theorem (CRT) with *p* and *q*. Now, *S* retrieves the tuple 〈*ID*_*i*_, $c_{i}^{\prime }$, *b*_*i*_〉, which is indexed by *ID*_*i*_, from the database and compares whether $c_{i}^{\prime }>c_{i}$ holds. If it is incorrect, *S* terminates the session. Otherwise, *S* updates the tuple 〈*ID*_*i*_, $c_{i}^{\prime }$, *b*_*i*_〉 to 〈*ID*_*i*_, *c*_*i*_, *b*_*i*_〉 and continues to the next step.
**Step 2**. Now, *S* computes $A_{i}^{\prime }$ = *H*(*ID*_*i*_∣∣*K*∣∣*b*_*i*_) and verifies whether $A_{i}^{\prime }=A_{i}$ holds. If it is incorrect, *S* terminates the session, otherwise accepts the login message *M*_1_ and authenticates *U*_*i*_.
**Step 3**. *S* selects a number $b\;\in_{R}\;Z_{n}^{*}$ and computes *d* = *a*⊕*b*, the session key *SK* = *H*(*ID*_*i*_∣∣*a*∣∣*b*∣∣*A*_*i*_) shared with *U*_*i*_ and *M*_2_ = *H*(*ID*_*i*_∣∣*A*_*i*_∣∣*d*∣∣*SK*). Then *S* delivers the message {*d*, *M*_2_} to *U*_*i*_ over a public network.
**Step 4**. On receiving {*d*, *M*_2_}, *U*_*i*_ computes *b* = *d*⊕*a*, the session key *SK* = *H*(*ID*_*i*_∣∣*a*∣∣*b*∣∣*A*_*i*_) and $M_{2}^{\prime }$ = *H*(*ID*_*i*_∣∣*A*_*i*_∣∣*d*∣∣*SK*). If $M_{2}^{\prime }\neq M_{2}$, *U*_*i*_ terminates the session. Otherwise, *U*_*i*_ authenticates *S* and accepts *SK* as the correct session key shared with *S*.

### 5.4 Password Change Phase

In the password change phase, the smart card is allowed to independently (i.e., without any assistance of *S*) change *U*_*i*_’s old password *PW*_*i*_ to the new password $PW_{i}^{n}$. We described the password change phase with the following steps:

**Step 1**. *U*_*i*_ inserts the smart card into the specific device and then keys his/her 〈*ID*_*i*_, *PW*_*i*_〉 into the smart card. The smart card then computes *B*_*i*_ = *H*(*ID*_*i*_∣∣*PW*_*i*_), *A*_*i*_ = *C*_*i*_⊕*B*_*i*_ and $D_{i}^{\prime }$ = *H*(*A*_*i*_∣∣*B*_*i*_). The smart card aborts the password change if $D_{i}^{\prime }\neq D_{i}$ holds. Otherwise, the smart card executes the next step.
**Step 2**. The smart card computes $B_{i}^{n}$ = $H(ID_{i}||PW_{i}^{n})$, $C_{i}^{n}$ = $A_{i}⊕B_{i}^{n}$ and $D_{i}^{n}$ = $H(A_{i}||B_{i}^{n})$. Now, the smart card updates the tuple 〈*H*(⋅), *n*, *C*_*i*_, *D*_*i*_, *c*_*i*_〉 to tuple 〈*H*(⋅), *n*, $C_{i}^{n}$, $D_{i}^{n}$, *c*_*i*_〉 into the memory.

### 5.5 Stolen/Lost Smart Card Revocation Phase

This phase is designed to issue a new smart card if *U*_*i*_ lost his/her old smart card. The description of this phase includes the following steps:

**Step 1**. The user *U*_*i*_ sends the smart card revocation request with his/her identity *ID*_*i*_ to *S* over a secure channel.
**Step 2**. *S* verifies the correctness of the identity *ID*_*i*_. If it is invalid, *S* terminates the request. Otherwise, *S* selects a new number $b_{i}^{\prime }\;\in_{R}\;Z_{n}^{*}$ and then computes $A_{i}^{\prime }=H(ID_{i}||K||b_{i}^{\prime })$. *S* updates the tuple 〈*ID*_*i*_, *c*_*i*_, *b*_*i*_〉 to 〈*ID*_*i*_, *c*_*i*_, $b_{i}^{\prime }\rangle$ into his/her database. Now, *S* writes the information 〈*H*(⋅), *n*, $A_{i}^{\prime }$, *c*_*i*_〉 into a new smart card and delivers it to *U*_*i*_ through a secure channel.
**Step 3**. On receiving the new smart card, *U*_*i*_ inserts it into the card reader and inputs his/her login identity *ID*_*i*_ and the new password $PW_{i}^{\prime }$ into the smart card. Then the smart card computes $B_{i}^{\prime }$ = $H(ID_{i}||PW_{i}^{\prime })$, $C_{i}^{\prime }$ = $A_{i}^{\prime }⊕B_{i}^{\prime }$ and $D_{i}^{\prime }$ = $H(A_{i}^{\prime }||B_{i}^{\prime })$. Now the smart card deletes the information $\langle A_{i}^{\prime }$, $B_{i}^{\prime }\rangle$ form the smart card and then updates the smart card’s memory with the tuple 〈*H*(⋅), *n*, $C_{i}^{\prime }$, $D_{i}^{\prime }$, *c*_*i*_〉.

The complete description of the Login and Authentication phases of our user authentication scheme is further presented in Table 3.

**Table 3:** Login and authentication phases of the proposed user authentication scheme.

**User *U*_*i*_/Smartcard**		**Server *S***
**User *U*_*i*_**:		
Insert ⟨*ID*_*i*_, *PW*_*i*_⟩		
**Smartcard**:		
Compute *B*_*i*_ = *H*(*ID*_*i*_∣∣*PW*_*i*_), *A*_*i*_ = *C*_*i*_⊕*B*_*i*_		
Compute $D_{i}^{\prime }$ = *H*(*A*_*i*_∣∣*B*_*i*_)		
If ($D_{i}^{\prime }\neq D_{i}$)		
terminate the session		
Else		
compute *c*_*i*_ = *c*_*i*_ + 1		
Choose $a\;\in_{R}\;Z_{n}^{*}$		
Compute *M*_1_ = (*ID*_*i*_∣∣*A*_*i*_∣∣*a*∣∣*c*_*i*_)^2^ mod *n*		
	$\overset{M_{1}}{\underset{\left(\text{via a public channel}\right)}{\to }}$	
		Obtain *ID*_*i*_, *A*_*i*_, *a*, *c*_*i*_ from *M*_1_
		Retrieve ⟨*ID*_*i*_, $c_{i}^{\prime }$, *b*_*i*_⟩ from database
		If ($c_{i}^{\prime }\leq c_{i}$)
		terminate the session
		Else
		update ⟨*ID*_*i*_, $c_{i}^{\prime }$, *b*_*i*_⟩ to ⟨*ID*_*i*_, *c*_*i*_, *b*_*i*_⟩
		Compute $A_{i}^{\prime }$ = *H*(*ID*_*i*_∣∣*K*∣∣*b*_*i*_)
		If ($A_{i}^{\prime }\neq A_{i}$)
		terminate the session
		Else
		select $b\;\in_{R}\;Z_{n}^{*}$
		Compute *d* = *a*⊕*b* and
		Session key *SK* = *H*(*ID*_*i*_∣∣*a*∣∣*b*∣∣*A*_*i*_)
		Compute *M*_2_ = *H*(*ID*_*i*_∣∣*A*_*i*_∣∣*d*∣∣*SK*)
	$\overset{\left\{d,M_{2}\right\}}{\underset{\left(\text{via a public channel}\right)}{\leftarrow }}$	
**Smartcard**:		
Compute *b* = *d*⊕*a* and		
Session key *SK* = *H*(*ID*_*i*_∣∣*a*∣∣*b*∣∣*A*_*i*_)		
Compute $M_{2}^{\prime }$ = *H*(*ID*_*i*_∣∣*A*_*i*_∣∣*d*∣∣*SK*)		
If ($M_{2}^{\prime }\neq M_{2}$)		
terminate the session		
Else		
authenticate *S* and		
Accept *SK* as session key		

## 6 Security and Functionality Analysis of the Proposed Scheme

This section is designed to prove the security and functionality strengths of our proposed scheme [46–48]. Now, we described the following assumptions about the attack capability of active and passive adversaries:

- The adversary A controls the communication channel [49, 50] i.e., he/she may intercept, block, inject, remove, or modify, any messages transmitted over the public media, in other words, all the messages communicated between *U*_*i*_ and *S* are transmitted via A.
- A may either (i) theft *U*_*i*_’s smart card and obtain the secret data from it through monitoring the timing information, power consumption and reverse engineering techniques which are proposed in [40–42] and try to obtain *U*_*i*_’s correct password in any off-line manner; or (ii) obtain *U*_*i*_’s password directly by some means. However, A cannot do both (i) and (ii) [37, 38].

Based on the aforesaid assumptions, the following theorems have been stated and proved against the proposed user authentication scheme.

**Theorem 1**. The proposed user authentication scheme could provide the user anonymity and user unlinkability.

*Proof*. Users’ anonymity or secrecy, i.e., the protection of user’s identity from the adversary is a great concern in many internet applications including integrated EPR information system, telecare medical information system (TMIS), online order placement, Pay-TV, wireless communications, banking transactions, etc [51–53]. The anonymity means that an adversary A cannot figure out the real identity *ID*_*i*_ of *U*_*i*_ from the eavesdropped authentication messages *M*_1_ and {*d*, *M*_2_}. Suppose that A captures *U*_*i*_’s authentication message *M*_1_ = (*ID*_*i*_∣∣*A*_*i*_∣∣*a*∣∣*c*_*i*_)^2^ mod *n* for a session. However, A cannot retrieve the identity *ID*_*i*_ from *M*_1_ due to the difficulties of quadratic residue problem and from *M*_2_ due to the one-way property of the hash function *H*(⋅). On the other hand, A cannot link that the two authentication messages *M*_1_ = (*ID*_*i*_∣∣*A*_*i*_∣∣*a*∣∣*c*_*i*_)^2^ mod *n* and $M_{1}^{\prime }$ = ${\left(ID_{i}||A_{i}||a^{\prime }||c_{i}^{\prime }\right)}^{2}$ mod *n* belong to the same user *U*_*i*_ and as a result the proposed scheme satisfies user anonymity and unlinkability [38, 45, 54].

**Theorem 2**. The proposed user authentication scheme could provide the perfect forward secrecy of the session key.

*Proof*. The perfect forward secrecy [43, 45, 55] ensures that a session key derived in a session will remains undisclosed even if the server’s secret key is compromised. In the proposed scheme, the session key is computed as *SK* = *H*(*ID*_*i*_∣∣*a*∣∣*b*∣∣*A*_*i*_), where *A*_*i*_ = *H*(*ID*_*i*_∣∣*K*∣∣*b*_*i*_) and the random numbers *a* and *b* are chosen by *U*_*i*_ and *S*, respectively. Therefore, even if A has the knowledge of secret key *K* of *S*, A needs to be extract *a* and *b* from *M*_1_ = (*ID*_*i*_∣∣*A*_*i*_∣∣*a*∣∣*c*_*i*_)^2^ mod *n* and {*d* = *a*⊕*b*, *M*_2_ = *H*(*ID*_*i*_∣∣*A*_*i*_∣∣*d*∣∣*SK*)} to derive the session key *SK*, however this is infeasible due to the quadratic residue problem [3, 35]. Thus, our scheme provides the functionality of session key perfect forward secrecy.

**Theorem 3**. The proposed user authentication scheme could resist the replay attack.

*Proof*. In replay attack, the adversary A captured a valid login message of previous session and then fraudulently replayed to current session to impersonate *U*_*i*_ or *S*. Assume that, in our scheme, A captured the previous login message *M*_1_ = (*ID*_*i*_∣∣*A*_*i*_∣∣*a*∣∣*c*_*i*_)^2^ mod *n* of *U*_*i*_ and replays it in the current session. However, *S* quickly detects that *M*_1_ is a replay message by comparing the counter *c*_*i*_ in the message *M*_1_ with the counter $c_{i}^{\prime }$ retrieves from *S*’s database. When *U*_*i*_ sends *M*_1_ = (*ID*_*i*_∣∣*A*_*i*_∣∣*a*∣∣*c*_*i*_)^2^ mod *n* to *S*, then *S* verifies it and stores the counter *c*_*i*_ to the tuple 〈*ID*_*i*_, *c*_*i*_, *b*_*i*_〉. Now, if the same *M*_1_ is replayed by the adversary in future session then the computed counter *c*_*i*_ is equal to or less than the retrieved counter *c*_*i*_. The counter *c*_*i*_ helps *S* to detect the replay attack [3]. Thus, the proposed user authentication scheme avoids the replay attack.

**Theorem 4**. The proposed user authentication scheme could resist the modification/forgery attack.

*Proof*. In the login phase, *U*_*i*_ sends *M*_1_ = (*ID*_*i*_∣∣*A*_*i*_∣∣*a*∣∣*c*_*i*_)^2^ mod *n* to *S* and *S* responds with the message {*d* = *a*⊕*b*, *M*_2_ = *H*(*ID*_*i*_∣∣*A*_*i*_∣∣*d*∣∣*SK*)} to *U*_*i*_ over an open channel. Since *A*_*i*_ is protected in *M*_1_ based on the difficulty of solving the QRP and in *M*_2_ by the one-way property of the hash function *H*(⋅), any modification of *M*_1_ and *M*_2_ by the adversary A will be detected by *U*_*i*_ and *S* through the verification equations $c_{i}^{\prime }>c_{i}$, $A_{i}^{\prime }=A_{i}$ and $M_{2}^{\prime }=M_{2}$. Therefore, our authentication scheme protects this kind of modification/forgery attack.

**Theorem 5**. The proposed user authentication scheme could resist the privileged-insider attack.

*Proof*. To make the convenient access of different application servers, user generally registers himself/herself by the common login identity and passwords. It is harmful for the user that if the password is compromised to the privileged-insider of a server, then he/she can easily impersonate the user and can login to other applications. In the registration phase of our scheme, *U*_*i*_ only sends his/her login identity *ID*_*i*_ not any password to *S*. Upon receiving the smart card from *S*, *U*_*i*_ inserts his/her *PW*_*i*_ into the smart card. As a result, *PW*_*i*_ is not exposed to the privileged-insider of *S*. Therefore, our scheme withstands the privileged-insider attack.

**Theorem 6**. The proposed user authentication scheme could provide the off-line password guessing attack from lost/stolen smart card.

*Proof*. The off-line password guessing attack is infeasible in our scheme. Assume that 𝓐 obtains *U*_*i*_’s smart card and extracts the parameters 〈*H*(⋅), *n*, *C*_*i*_, *D*_*i*_, *c*_*i*_〉, where *C*_*i*_ = *H*(*ID*_*i*_∣∣*K*∣∣*b*_*i*_)⊕*H*(*ID*_*i*_∣∣*PW*_*i*_) and *D*_*i*_ = *H*(*H*(*ID*_*i*_∣∣*K*∣∣*b*_*i*_)∣∣*H*(*ID*_*i*_∣∣*PW*_*i*_)). Now A may try to guess the correct password *PW*_*i*_ of *U*_*i*_ in off-line processes. However, without knowing *K* and *b*_*i*_, A cannot find *PW*_*i*_. Thus, our user authentication scheme strongly resists the off-line password guessing attack from lost/stolen smart card.

**Theorem 7**. The proposed user authentication scheme could resist the ephemeral secret leakage attack.

*Proof*. This attacks states that the none of the session keys should be compromised with the disclosures of session random numbers (ephemeral secrets) [56]. The ephemeral secrets may be compromised [37, 45] and it is quite common in real environments due to the following reasons: (i) user and server depended on the internal/external source of random number generator which may be controlled by A and (ii) the random numbers are generally stored in insecure device. If the random numbers aren’t erased properly in each session, A may hijack users’ computer and learn the random numbers. In our scheme, *U*_*i*_ and *S* generate the session key as *SK* = *H*(*ID*_*i*_∣∣*a*∣∣*b*∣∣*A*_*i*_), where *A*_*i*_ = *H*(*ID*_*i*_∣∣*K*∣∣*b*_*i*_). Suppose that 〈*a*, *b*〉 is disclosed and A knows it. However, A cannot compute *SK* without *A*_*i*_. Therefore, the ephemeral secret leakage attack is infeasible in the proposed scheme.

**Theorem 8**. The proposed user authentication scheme could resist the known-key attack.

*Proof*. This attack states that, none of the session keys are compromised even if the adversary A knows some other session keys [43]. In our scheme, *U*_*i*_ and *S* establish a session key *SK* = *H*(*ID*_*i*_∣∣*a*∣∣*b*∣∣*A*_*i*_), where *A*_*i*_ = *H*(*ID*_*i*_∣∣*K*∣∣*b*_*i*_). The numbers *a* and *b* are randomly chosen from $Z_{n}^{*}$ and hence *SK* is also random and independent in each session. Therefore, with the knowledge of previous session keys A cannot compute a new session key. Accordingly, the known-key attack is impossible in our user authentication scheme.

**Theorem 9**. The proposed user authentication scheme could resist the unknown key-share attack and provide the explicit key confirmation property of the agreed session key.

*Proof*. The unknown key-share attack [43] is a situation that *U*_*i*_ finishes the session by believing that he/she shares the session key *SK* correctly with *S*, however, *S* mistakenly believes that *SK* is instead shared with the adversary A. In the proposed scheme, *S* computes the session key *SK* after validating the messages *M*_1_. To validate the message {*d*, *M*_2_} and to get the confirmation about the agreed session key *SK*, *U*_*i*_ computes *SK* = *H*(*ID*_*i*_∣∣*a*∣∣*b*∣∣*A*_*i*_), $M_{2}^{\prime }$ = *H*(*ID*_*i*_∣∣*A*_*i*_∣∣*d*∣∣*SK*) and authenticates *S* and accepts *SK* as the correct session key if the condition $M_{2}^{\prime }=M_{2}$ hold. Therefore, *U*_*i*_ and *S* mutually authenticate each other and then compute the session key *SK*, accordingly our scheme enjoys the unknown key-share attack resilience and explicit key confirmation of the session key.

**Theorem 10**. The proposed user authentication scheme could provide efficient and user friendly password change option.

*Proof*. Our scheme gives the flexibility to the user to choose low-entropy password by himself/herself and change the password periodically without remote server’s assistance [28, 29]. Moreover, the proposed scheme detects the wrong password and identity during the login phase and password change phase. In these processes, if *U*_*i*_ keys either wrong password $PW_{i}^{*}$ or identity $ID_{i}^{*}$ by mistake, the smart card reports the error message to *U*_*i*_ without any consultation with *S* [37]. On the other hand, if A thefts *U*_*i*_’s smart card, however, he/she does not have the capability to update smart card’s memory without correct password *PW*_*i*_, and consequently the denial of service (DoS) attack is eliminated in our scheme. If A tries to do the same with wrong password, the smart card will be locked immediately if the number of login failure exceeds the pre-defined limit.

**Theorem 11**. The proposed user authentication scheme could provide mutual authentication and session key agreement between the user and the remote server.

*Proof*. In our scheme, *S* authenticates *U*_*i*_’s login message *M*_1_ = (*ID*_*i*_∣∣*A*_*i*_∣∣*a*∣∣*c*_*i*_)^2^ mod *n* by verifying the conditions $c_{i}^{\prime }>c_{i}$ and $A_{i}^{\prime }=?A_{i}$. Similarly, *U*_*i*_ verifies *S*’s response message 〈*d*, *M*_2_〉 by validating whether $M_{2}^{\prime }=M_{2}$ holds. Without 〈*PW*_*i*_, *K*〉, A cannot impersonate none of *U*_*i*_ and *S*. Hence, the secure mutual authentication between *U*_*i*_ and *S* is achieved in our scheme. Moreover, after mutual authentication, *U*_*i*_ and *S* compute a random and unique session key *SK* = *H*(*ID*_*i*_∣∣*a*∣∣*b*∣∣*A*_*i*_), where *A*_*i*_ = *H*(*ID*_*i*_∣∣*K*∣∣*b*_*i*_).

## 7 Performance Analysis of the Proposed Scheme

In the following, we have performed the comparison analysis of our authentication scheme with the schemes proposed in [3, 34, 57–59]. Here, the following notations are described for this purpose:

- *t*_*h*_ : Time needed to execute a hash function.
- *t*_*m*_ : Time needed to execute a modular squaring computation.
- *t*_*q*_ : Time needed to execute a square root operation with the modulus *n*.

The comparative result of the proposed scheme and the schemes in [3, 34, 57–59] from the aspects of computation cost and communication round is listed in the Table 4. Our scheme proposed all the required phases where the schemes in [3, 34, 57–59] do not have password change phase and smartcard revocation phase. Furthermore, we observed that our scheme is more robust and computation and communication cost efficient than the schemes devised in [3, 34, 57–59].

**Table 4:** Computation cost comparison of the proposed user authentication scheme with others.

Attributes	Wen [3]	Zhu [34]	Wu et al. [57]	Cheng et al. [58]	Lee [59]	Proposed
*A*_1_	2*t*_*h*_ + *t*_*m*_	2*t*_*h*_ + *t*_*m*_	2*t*_*h*_ + *t*_*m*_	*t*_*h*_	2*t*_*h*_	2*t*_*h*_
*A*_2_	9*t*_*h*_ + *t*_*q*_	5*t*_*h*_ + *t*_*q*_	6*t*_*h*_ + *t*_*m*_ + 2*t*_*q*_	12*t*_*h*_ + *t*_*m*_ + *t*_*q*_	8*t*_*h*_ + *t*_*m*_ + 2*t*_*q*_	5*t*_*h*_ + *t*_*m*_ + *t*_*q*_
*A*_3_	4*t*_*h*_	4*t*_*h*_ + *t*_*m*_	NA	NA	NA	4*t*_*h*_
*A*_4_	NA	NA	NA	NA	NA	3*t*_*h*_
*A*_5_	2	3	2	2	2	2

*A*_1_: Computation cost in registration phase, *A*_2_: Computation cost in login phase and authentication phase, *A*_3_: Computation cost in password change phase, *A*_4_: Computation cost in smartcard revocation phase, *A*_5_: Number of message communications, **NA**: Not applicable (this pase is not proposed by the author (s)).

**Fig 1:**
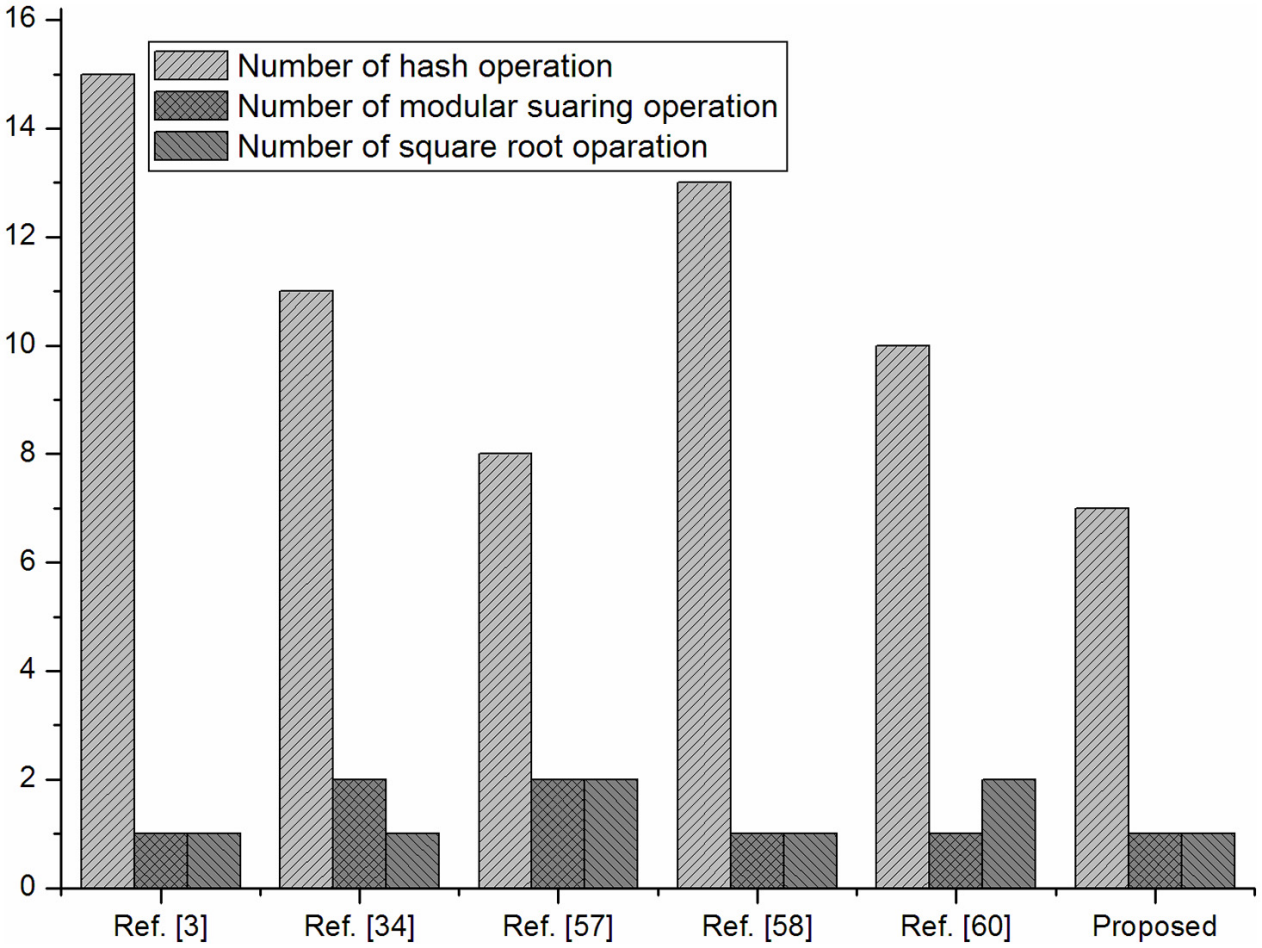
Number of hash, modular squaring and square root operations for registration and login phases.

We also given a comparison in Fig 1 against the number of operations used in the registration and login phased of the schemes in [3, 34, 57–59] with the proposed scheme.

In 2011, based on QRP, Wu et al [57] proposed a user authentication scheme using smartcard. However, the scheme is vulnerable to (1) privileged-insider attack since the plaintext password is sending to the server for registration, (2) the scheme does not verify the keyed password and identity in the login phase, (3) the scheme does not have password change phase, (4) it has no provision to revoke the lost/stolen smart card, (5) no session key agreement method is proposed and (6) user anonymity and unlinkability are violated as the identity is transmitted in plaintext form. In the year 2012, in order to ensure users’ privacy, Zhu [34] proposed a user authentication schemes for telecare medicine information systems. We carefully observed that the Zhu’s scheme [34] is not free from attack since (1) the scheme does not verify the correctness of the login identity and password in the login phase, (2) the scheme does not verify the correctness of the login identity and password in the password change phase, (3) the scheme has no provision to revoke the smartcard in case if the smartcard is stolen or lost, (4) the scheme does not design a session key agreement method during login and authentication phases, (5) user anonymity and user untracibility are not present in this scheme. In 2013, Cheng et al. [58] proposed a a biometric-based remote user mutual authentication and session key agreement scheme using QRP. However, Yoon [60] showed that the scheme is insecure from the stolen smart card attack, server spoofing attack and does not provide session key forward secrecy. We also observed that the scheme does not have provision for password change and lost/stolen smart card revocation. In addition, the scheme does not verify the correctness of keyed identity and password in the login phase. Further, the scheme is also suffered from the ephemeral secrets leakage attack as the session key solely depended on the random numbers chosen by the user and server. In 2015, Lee [59] proposed an efficient smartcard-based two-factor remote user mutual authentication scheme. However, we observed that the scheme is not secure since (1) the scheme does not proposed any password change method, (2) the scheme does not proposed any lost/stolen smart card revocation method, (3) The scheme has no provision for session key agreement during login and authentication phases, (4) the scheme does not verify the keyed password and identity in the login phase, (5) the privileged-insider attack since the plaintext password is sending to the server for registration.

In the comparative analysis of the proposed scheme and the schemes [3, 34, 57–59] with respect to security and functionality are included in the Table 5. Form Tables 4 and 5, it can be see that the proposed user authentication scheme includes more security and functional features compared to [3, 34, 57–59].

**Table 5:** Security and functionality comparison of the proposed scheme with other existing schemes.

Attributes	Wen [3]	Zhu [34]	Wu et al. [57]	Cheng et al. [58]	Lee [59]	Proposed
*F*_1_	No	No	No	No	No	Yes
*F*_2_	No	No	NA	NA	NA	Yes
*F*_3_	No	Yes	Yes	No	Yes	Yes
*F*_4_	No	Yes	No	Yes	No	Yes
*F*_5_	No	No	No	No	No	Yes
*F*_6_	No	NA	NA	No	NA	Yes
*F*_7_	No	NA	NA	Yes	NA	Yes
*F*_8_	No	Yes	NA	No	No	Yes
*F*_9_	Yes	NA	NA	No	No	Yes
*F*_10_	Yes	No	No	Yes	Yes	Yes
*F*_11_	Yes	Yes	Yes	Yes	Yes	Yes
*F*_12_	Yes	Yes	Yes	Yes	Yes	Yes
*F*_13_	Yes	Yes	NA	No	Yes	Yes
*F*_14_	Yes	Yes	Yes	Yes	Yes	Yes

*F*_1_: Login identity and password detection in the login phase; *F*_2_: Login identity and password detection in the password change phase; *F*_3_: Impersonation attack is avoided; *F*_4_: Privileged-insider attack is avoided; *F*_5_: Lost/stolen smart card revocation phase is present; *F*_6_: Explicit session key confirmation property is present; *F*_7_: No key control property is present; *F*_8_: Password is changed without any help from the server; *F*_9_: Ephemeral secrets leakage attack is avoided; *F*_10_: User anonymity and unlinkability are present; *F*_11_: Password guessing attack from lost smart card is avoided; *F*_12_: Replay attack is avoided; *F*_13_: Forward secrecy of the session key is present; *F*_14_: modification/forgery attack is avoided.

## 8 Conclusions

The privacy and confidentiality of patient information and the untraceability and anonymity of patient have considered important factors from the security research communities for any user authentication system used in different healthcare applications. In keeping with these requirements, Wen proposed an enhanced user authentication scheme against Lee et al.’s authentication scheme for the EPR information system. However, this paper analyzed Wen’s scheme and demonstrated that it has many security and design problems and thus, it may not be considered appropriate for the secure and efficient healthcare applications. We have then taken into consideration the intractability assumption of the quadratic residue problem in the multiplicative group and proposed another two-factor user authentication scheme with more security and functionality aspects than existing schemes.
